# Integrating donor conception into identity development: adolescents in fatherless families

**DOI:** 10.1016/j.fertnstert.2016.02.033

**Published:** 2016-07

**Authors:** Jenna Slutsky, Vasanti Jadva, Tabitha Freeman, Sherina Persaud, Miriam Steele, Howard Steele, Wendy Kramer, Susan Golombok

**Affiliations:** a Center for Attachment Research, New School for Social Research, The New School, New York, New York; b Centre for Family Research, University of Cambridge, Cambridge, United Kingdom; c Donor Sibling Registry, Nederland, Colorado

**Keywords:** Adolescence, attachment, donor conception, identity, sperm donation

## Abstract

**Objective:**

To study the processes by which donor-conceived children incorporate donor conception into their subjective sense of identity.

**Design:**

Cross-sectional.

**Setting:**

Family homes.

**Patient(s):**

Nineteen donor-conceived adolescents.

**Intervention(s):**

Administration of an interview and questionnaire.

**Main Outcome Measure(s):**

The mother-child relationship was assessed through the Friends and Family Interview, a semistructured interview designed to assess adolescents' security of attachment in terms of secure-autonomous, insecure-dismissive, insecure-preoccupied, and insecure-disorganized attachment patterns. The Donor Conception Identity Questionnaire assessed adolescents' thoughts and feelings about donor conception, yielding two factors: [1] curiosity about donor conception and [2] avoidance of donor conception.

**Result(s):**

Statistically significant associations were found between the Curiosity scale and the secure-autonomous and insecure-dismissing attachment ratings. Adolescents with secure-autonomous attachment patterns were more interested in exploring donor conception whereas those with insecure-dismissing patterns were less likely to express curiosity. Insecure-disorganized attachment ratings were statistically significantly correlated with the Avoidance scale, indicating higher levels of negative feelings about donor conception.

**Conclusion(s):**

The results of this study of the influence of parent-child relationships on thoughts and feelings about donor conception in adolescence suggest that the valence of the parent-child relationship influences adolescents' appraisal of their donor conception within the context of their growing sense of identity.

**Discuss:** You can discuss this article with its authors and with other ASRM members at **http://fertstertforum.com/slutskyj-integrating-donor-conception-identity/**

Research on the diverse family forms made possible by donor conception has largely focused on comparisons between family types. These studies have generally shown that donor-conceived families headed by heterosexual couples, lesbian couples, and single heterosexual mothers do not differ in terms of family functioning or child adjustment from heterosexual two-parent families formed without medical assistance 1, 2. There is little research on variation within donor-conceived families, particularly in relation to the internal processes by which donor-conceived children incorporate information regarding their donor conception into their developing sense of identity. The question of how a child develops an understanding of donor conception becomes particularly relevant at adolescence when issues surrounding identity formation and individuation become salient (3).

Research on the identity formation of adopted adolescents offers a useful starting point for exploring identity development in donor conception 4, 5. Based on Erikson's 6, 7 theory of identity development, which views adolescence as a time of great productivity in identity formation, Grotevant and Cooper (8) examined the process of adolescent identity development within the familial context, particularly in relation to adoptive families. They argued that to develop a secure sense of identity adolescents must feel safe and sufficiently connected to explore their nascent independence. They begin to make active choices, such as selecting a career path, at the same time as making meaning out of those aspects of themselves they did not choose, such as having been adopted (9). As part of this process of “meaning-making,” some adopted adolescents seek information about their birth family (10). Similar processes have been reported for families created through donor conception. Some donor-conceived adolescents search for information about their donor and donor siblings out of curiosity and also to enhance their developing sense of identity 11, 12, 13, 14.

Adopted adolescents' curiosity about their family of origin can be hindered by perceived barriers (15), such as parents who are thought to be discouraging of the process. However, parents are also uniquely positioned to serve in a facilitating capacity through a willingness to explore adoption-related issues 16, 17. In a longitudinal study of adoptive families from adolescence to early adulthood, Von Korff and Grotevant (18) found that more frequent adoption-related conversations promoted a more coherent adoptive identity narrative. It was concluded that ongoing conversations within adoptive families assist in narrative building, thus helping adopted children make sense of the past.

Given the mutual emphasis on narrative development, attachment theory provides a useful theoretical bridge for extending adoption identity theories to donor conception. According to attachment theory 19, 20, the early relationship with parents underpins the development of internalized mental representations, thus influencing a child's personality development, perceptions, and social interactions throughout the developmental trajectory. Securely attached children conceptualize the parent(s) as a secure base and safe haven available for protection and support should the attachment system be activated, for example, by a threatening situation. These secure internal working models serve as protective factors as the child negotiates developmental challenges (21). Conversely, insecure attachment patterns, often a result of unpredictable or chaotic early experiences with parents, are associated with multiple and diverse negative outcomes ranging from affect regulation difficulties to dissociation (22).

As children enter adolescence, increasingly sophisticated metacognitive abilities allow them to positively and negatively evaluate their attachment figures as they simultaneously develop their own perspective on, or narrative about, their attachment to their parent(s) 23, 24. It is at this point that attachment patterns emerge in autobiographical narratives. These patterns are indicative of a combination of internal working models, reflective functioning, and adaptive functioning skills used to navigate the developmental challenges associated with adolescence, including identity development. Coherence and evidence of an adaptive response to stressors are critical components of a secure narrative (25). Echoing Grotevant and colleagues' theories of adoptive identity development 8, 9, 10, 18, attachment theory suggests that it is not necessarily the quality of one's interpersonal experiences that influences internal working models and attachment patterns, but rather the meaning that one is able to construct out of such experiences.

Although potentially beneficial to the process of meaning-making and identity development, engaging with various aspects of donor conception, such as searching for donor relations and initiating conversations about genetic origins, can be perceived as a threatening and intimidating process by both parents and children in donor-conceived families 13, 26. Given the value of safety within interpersonal relationships (27), it is reasonable to expect that a child who has internalized their parent(s) as consistently supportive, even under stressful or threatening circumstances, is more likely to trust that the parent(s) can scaffold the exploration of their donor conception. It is thus hypothesized that, as donor-conceived adolescents navigate the demands of identity formation, those who have developed secure internal working models of their parental relationship(s) will feel more comfortable with the process of positively integrating donor conception into a coherent sense of identity.

The present study focused on the adolescent children of single mothers and lesbian couples conceived through anonymous sperm donation, as the children in these families are more likely than children in two-parent heterosexual families to have become aware of their donor conception at an early age 11, 12. In addition, studying the children of single mothers and lesbian couples enables the process of donor-conception identity development to be examined in the absence of the potentially confounding influence of a father in the home as donor-conceived adolescents in two-parent heterosexual families are less likely to explore their donor connections in order not to upset their parents (13).

## Materials and methods

### Participants

The participants were recruited through the Donor Sibling Registry, a U.S.-based registry that facilitates contact between same-donor offspring, their parents, and donors. In the first instance, an e-mail giving information about the study and requesting assistance was sent to single mothers and partnered lesbian mothers who [1] were living within the tristate area of New York designated for its accessibility to the researchers, [2] had one or more adolescent children conceived by donor insemination, and [3] had found at least one of their child's donor-siblings. Although 146 e-mails were sent out, it was not possible to determine how many mothers actually received or opened the initial e-mail. As the study was designed primarily as an in-depth qualitative study of adolescents' experiences of contact with their donor siblings (reported elsewhere), the aim was to recruit approximately 20 adolescents. The first 28 mothers to give permission for their contact details to be passed on to the researchers were contacted by one of the authors (J.S. or S.P.) to describe the study and request participation. Twenty-one mothers agreed for their family to take part, representing a participation rate of 78%. Nineteen adolescents conceived by donor insemination took part in this research, representing 90% of those who were approached about the study. Of the two adolescents who did not take part, one was not interested in participating, and one was attending school abroad. As face-to-face interviews were being conducted with donor-conceived adolescents, the study provided an opportunity to assess not only their relationships with donor siblings but also their attachment relationships with parents.

The adolescents ranged in age from 12 to 19 years (mean 14.18 ± 2.20 standard deviation). Two sets of two adolescents were from the same family. The mean age of the mothers was 52 years, all were white, and 14 (82%) had a bachelor's degree or higher qualification. The majority (15 participants; 78.9%) were employed full-time outside of the home. Fifteen (79%) of the adolescents were female, and 4 (21%) were male. All were enrolled in middle school or high school, with the exception of one who had graduated high school. All had been conceived by donor insemination using an anonymous sperm donor. Twelve adolescents (63%) were born to single mothers and 7 (37%) to lesbian couples. When asked when they had been told of their donor conception, 2 (10.5%) adolescents reported they had “always known,” 9 (47.4%) could not recall, and 8 (42.1%) reported they had been told at or before the age of 7. None had been in contact with their donor. However, all had located at least one donor sibling—that is, a half-sibling in another family born from the same donor as themselves.

### Procedure

One of two trained researchers (J.S. or S.P.) interviewed the adolescents in their homes. Written, informed consent was obtained from participants aged 18 years and over, and written parental consent and written and verbal assent were obtained from participants under the age of 18 years. Ethics approval for this study was obtained from both the University of Cambridge Psychology Research Ethics Committee and the New School's institutional review board. The researcher administered an audio-recorded, semistructured interview and a questionnaire to the adolescent in a private room. The interviews lasted approximately 1 hour.

### Measures

#### Friends and Family Interview

The adolescents took part in a modified version of the Friends and Family Interview (FFI) [(28); H. Steele et al., unpublished paper], a semistructured interview designed to assess adolescents' security of attachment. Information was obtained on the adolescent's relationship with their mother in single-mother families and with the mother who spent the most time with them in the lesbian-couple families. They were also asked about their coping strategies and their perceived social support systems including parents, friends, and others.

The interviews were coded according to the FFI Rating and Classification System [(25); Steele et al., unpublished paper]. Interview narratives were transcribed and coded on a number of dimensions such as coherence, reflective functioning skills, adaptive response, and perception of a parent as available for emotional support. The interviews were coded with an emphasis on the adolescents' coping strategies and the manner in which they discussed their relationship with their parent(s). The individual dimensions ultimately contributed to ratings of the following four attachment patterns, each rated on a 4-point scale ranging from 1 (no evidence) through 2 (mild evidence) to 3 (moderate evidence) and 4 (marked evidence): (1) secure-autonomous, (2) insecure-dismissing, (3) insecure-preoccupied, and (4) insecure-disorganized. These attachment patterns are considered to be central indicators of the internal working models a child has acquired based on early experiences with their caregiver(s) 19, 25. Each attachment pattern was rated individually as the FFI relies on a dimensional rather than categorical approach (25). Thus, a score ranging from 1 to 4 was obtained for each of the four scales for each adolescent.

Attachment pattern codes were each rated by a trained coder according to a set of criteria relating to both the narrative content and process. Ratings of secure-autonomous attachment patterns in FFI narratives are associated with high coherence, high adaptive coping, the capacity for needing others and exploring important relationships, flexibility to change views on others and events, an ease with imperfections of self and others, and an acceptance of the failings of parents and family members. Ratings of insecure-dismissing patterns correspond with a portrayal of the self as strong, minimal expression of hurt feelings, minimization of negative experiences, abstract description of experiences, a focus on concrete elements of relationships, and either idealization of parents or an emphasis on the negative aspects of parent behavior. Ratings of insecure-preoccupied patterns are associated with high levels of anger and characterized by rote responses persistently tied to parents, oscillation in evaluation of parents, and excessive blaming of parents or self. Ratings of insecure-disorganized patterns are associated with low narrative coherence, high derogation of self, contradictory strategies, dissociated states of mind, and references to frightening experiences that remain unresolved (Steele et al., unpublished paper).

#### Donor Conception Identity Questionnaire

The Donor Conception Identity Questionnaire (DCIQ) was designed by the authors (Jadva, Freeman, and Golombok) to assess the manner in which adolescents had integrated knowledge of donor conception into their subjective sense of identity. The items were informed by previous investigations of donor conception and adoption 8, 29, 30, 31. The adolescents rated their feelings about being donor conceived, their willingness to discuss donor conception with others, the frequency and quality of their thoughts about the donor and donor conception, the level of importance they ascribed to being donor conceived, and the extent to which they had considered their donor and donor conception in the context of “who they are.” Each item was rated on a 5-point scale ranging from 1 (“strongly agree”) to 5 (“strongly disagree”).

A principal component analysis with varimax rotation was conducted on the original 25 items of the DCIQ. Items with cross-loadings above 0.30 (n = 7 items) and items that only loaded negatively (n = 2 items) were excluded. As shown in Table 1, a model was identified in which the adolescents' thoughts and feelings about donor conception and identity loaded onto two distinct factors, each with eigenvalues >1. The model accounted for 63% of the variance. The two factors were described as [1] Curiosity about donor conception and [2] Avoidance of donor conception. The Curiosity factor reflected thinking about donor conception, a sense of flexibility and acceptance of donor conception, and positive feelings about donor conception. The items included “I have thought a great deal about donor conception,” “Being donor conceived is just part of who I am,” and “I think a lot about the characteristics I might share with my donor.” The Avoidance factor reflected a sense of disengagement from the topic and negative feelings, such as anger and anxiety, about being donor conceived. Examples of items are “I try to avoid the topic of donor conception because it raises a lot of questions,” “I feel embarrassed if others know I am donor conceived,” and “I like to keep my donor conception a secret.” The scores on the Curiosity and Avoidance scales represented the sum of the loadings of the items on the Curiosity and Avoidance factors, respectively. Cronbach alphas were 0.90 for Curiosity and 0.91 for Avoidance.

## Results

The majority of the sample (n = 12, 63.2%) was rated as higher on the secure-autonomous dimension than they were on the other three attachment dimensions, demonstrating dominant secure attachment patterns. In contrast, 7 (36.8%) demonstrated dominant-insecure attachment patterns, including five who were rated highest on the insecure-dismissing dimension, and two who were rated highest on the insecure-preoccupied dimension.

The statistical analyses were conducted in two stages. In the first stage, polyserial correlations were conducted to examine associations between the DCIQ and the FFI as the DCIQ produced scores on an interval scale and the FFI produced scores on an ordinal scale. Multiple linear regression was then used, where appropriate, to further examine significant associations between the DCIQ and the FFI.

Polyserial correlations were conducted between the Curiosity and Avoidance scales of the DCIQ and each of the attachment scales (secure-autonomous, insecure-dismissing, insecure-preoccupied, and insecure-disorganized). A statistically significant correlation was found between the Curiosity scale and the secure-autonomous (*r* = 0.53, *P*=.017) attachment rating, and a not statistically significant trend was found between the Curiosity Scale and the insecure-dismissing (*r* = −0.40, *P*=.085) attachment rating, reflecting higher levels of curiosity about, and acceptance of, donor conception among adolescents with higher levels of secure-autonomous attachment patterns and lower levels of insecure-dismissing attachment patterns. For the Avoidance scale, there was a statistically significant correlation with the insecure-disorganized attachment scale (*r* = 0.64, *P*=.003), indicating a higher level of avoidance of and negative feelings about donor conception among adolescents with higher levels of insecure-disorganized attachment patterns (Table 2).

Multiple linear regression was used to further investigate the relationship between secure-autonomous and insecure-dismissing attachment patterns and the adolescents' curiosity regarding their donor conception. The outcome variable was the factor score of Curiosity, and the predictors were the secure-autonomous and insecure-dismissing attachment scale scores. Adolescents' scores on these two attachment patterns were statistically significantly and negatively correlated (Spearman's rho = −0.61, *P*=.006). Therefore, to avoid multicollinearity, the regression model was conducted twice, once with the secure-autonomous attachment scale scores and once with the insecure-dismissing attachment scale scores. The model with secure-autonomous attachment as the predictor accounted for 21.0% of variance in adolescents' Curiosity [*F* (1,18) = 4.52, *P*=.048]. Higher ratings of secure-autonomous attachment were statistically significantly associated with higher levels of Curiosity (β = 0.46, *P*=.048). The model with insecure-dismissing attachment as the predictor accounted for 25.7% of variance in adolescents' Curiosity [*F* (1,18) = 5.89, *P*=.027]. Higher ratings of insecure-dismissing attachment were statistically significantly associated with lower levels of Curiosity (β = −0.51, *P*=.027).

## Discussion

Adolescents with secure-autonomous attachment patterns were more interested in exploring their donor conception whereas those with insecure-dismissing attachment patterns were less likely to express curiosity. This curiosity is important, given its potential value in the critical adolescent developmental task of investigating what aspects of self will be integrated (or not) into a subjective sense of identity 6, 7, 9, 32. In addition, adolescents with insecure-disorganized attachment patterns were more likely to express a preference for avoiding consideration of their donor conception. There were no statistically significant findings relating to insecure-preoccupied attachment patterns, possibly owing to the small sample size and limited within-group representation.

The relationship between attachment and expressed curiosity in donor conception may be understood through the points of convergence between Grotevant and Cooper's 9, 32 adoptive identity development theory and attachment theory 19, 20, 33. Adolescent adoption identity theory asserts that a co-occurring sense of connectedness to the family and a growing sense of independence facilitates exploration of adoption-related identity formation (10). This critical balance between autonomy and connectedness is consistent with fundamental characteristics of secure attachment in adolescence in which decreasing dependence on parents alongside an increasing willingness to explore the world scaffolds numerous developmental milestones 23, 34.

This complex balance between autonomy and connectedness is best served by mutual recognition by both parent and child of the need for communication as the family strives to accommodate the ultimately symbiotic but at times conflicting demands of connectedness and autonomy 23, 35. Given these conditions, it is perhaps not surprising that securely attached adolescents would be more willing to engage in the sometimes challenging task of exploring donor conception, which calls for independent assessment of an issue fundamentally linked to the parent. This task necessitates careful equilibrium between an openness to family connectedness and a growing desire to titrate the family influence on an increasingly independent intellectual and emotional life (34). Intrafamily communication, associated with security in adolescence, is not only a critical tool in maintaining and constantly renegotiating this delicate balance (36) but has also been found to facilitate the process of building a coherent sense of adoption identity (18).

In contrast, adolescents who demonstrated evidence of insecure attachment were more likely to show a preference for avoiding the topic of donor conception altogether. Those who demonstrated insecure-dismissive attachment were least likely to express curiosity. Dismissing attachment patterns in adolescence have been characterized by limited autonomy, less communication with parents (37), withdrawal and distraction from attachment-related cues 23, 38, and devaluing of close attachment relationships 20, 39. Given the inextricable link between attachment figures (parents) and donor conception, lack of engagement with the topic is consistent with the larger set of insecure-dismissing cognitive and relational strategies.

The minority of the sample who showed insecure-disorganized attachment patterns were most likely to feel negatively about, and avoid engagement with, the issue of their donor conception. Conclusions about the potential effect of disorganized attachment on donor-conception identity must be drawn carefully given the limited representation of these patterns in this sample. However, it is noteworthy that those adolescents who demonstrated evidence of disorganization, associated with negative psychological outcomes (22), were most likely to endorse negative feelings toward donor conception including anger, anxiety, and shame. Further investigation is needed to determine whether this finding is reflective of an etiologic explanation of negative feelings toward donor-conception origins.

The findings of this study must be interpreted with caution due to several limitations, particularly the small sample drawn from a narrow geographic region. Although the participation rate of families directly contacted by the researchers was high, it is not known how many mothers received or opened the initial e-mail from the Donor Sibling Registry. To the extent that biases were present in the sample, these are likely to reflect a greater engagement with the issue of donor conception and a greater inclination to search for donor siblings than would be found in the general population of donor-conception families. It should also be emphasized that the adolescents in the present study were all aware of their genetic origins. They had all been registered on the Donor Sibling Registry by their parents, most when very young. It is likely that children of parents who had not demonstrated such willingness to embrace the topic of their donor conception would have had a different experience of negotiating this aspect of identity development. Moreover, this study included children of single mothers and lesbian couples only. Curiosity about donor conception has been found to be higher in families where a father is not present 12, 40.

An advantage of the study is that it is the first to conduct in-depth interviews with donor-conceived adolescents. This made it possible to assess the adolescents' attachment relationships with their parents. The representation of both insecure and secure attachment patterns and both positive and negative feelings toward donor conception indicate that the sample was diverse enough to reveal associations between attachment security and donor-conception identity. All participants were living at home and thus in environments offering a similar opportunity for conversations with their parents. In spite of the small sample size and need for replication with a more representative sample, meaningful and potentially important associations were identified between attachment security and donor-conception identity, thus shedding light not only on differences in the experiences of donor-conceived adolescents but also on the psychological processes through which these differences arise.

Although the study was conducted with adolescents, it was designed to capture parental internal representations formed in childhood as they relate to adolescent identity development. Allen et al. (34) have highlighted the similarity between early attachment processes and parental representations in adolescence, likening the balance between connectedness and autonomy in adolescence to what Bowlby (41) called the secure-base phenomenon, in which a securely attached young child is willing to explore the unknown when they know their “secure base” is available should a threat arise. Although there have been conflicting findings on the stability of attachment from infancy to adolescence (42), internal working models associated with early attachment-related experiences are generally considered to remain relatively stable over time 20, 43, 44. Thus, the present study offers a preliminary insight into the influence of parent-child relationships in childhood on thoughts and feelings about donor conception in adolescence. The findings suggest that the valence of the parent-child relationship influences the adolescents' appraisal of their donor conception within the context of their growing sense of identity. Clinical implications of the findings point to the need for professionals working with these families to encourage parents to focus not only on whether to disclose the donor conception to their children but also to consider the wider emotional context of the family. The optimal emotional context from an attachment perspective is one in which openness is experienced in a balanced, supportive, and coherent manner.

## Figures and Tables

**Table 1:** Summary of principal component analysis for Donor Conception Identity Questionnaire (DCIQ) using varimax rotation.

Item	Factors	
Curiosity	Avoidance
I am still trying to figure out how donor conception relates to who I am.	0.764^a^	–
Being donor conceived makes me feel special.	0.711^a^	−0.240
I have thought a great deal about donor conception.	0.888^a^	–
After a conversation about donor conception I tend to feel upset.	0.251	0.564^a^
It's important for me to be in contact with other donor-conceived individuals.	0.747^a^	–
Being donor conceived is just part of who I am.	0.802^a^	–
I try to avoid the topic of donor conception because it raises a lot of questions.	–	0.880^a^
I feel angry that I am donor conceived.	–	0.601^a^
I think a lot about the characteristics I might share with my donor.	0.852^a^	0.289
Donor conception doesn't enter into my life or my decisions at all (reversed).	0.654^a^	0.226
I understand myself better because I have thought about who I am in relation to my parents and donor.	0.698^a^	–
I feel embarrassed if others know I am donor conceived.	–	0.882^a^
I like to keep my donor conception a secret.	–	0.864^a^
I am happy to tell anyone about my donor conception (reversed).	–	0.884^a^
I feel ashamed of being donor conceived.	−0.220	0.794^a^
I worry about being bullied or teased about being donor conceived.	–	0.736^a^
Percentage of variance	30.353	32.734

a Factor loadings above 0.40. Values below 0.20 are not included.

**Table 2:** Polyserial correlation coefficients for the Friends and Family Interview (FFI) ratings of attachment and the Curiosity and Avoidance scales of the Donor Conception Identity Questionnaire (DCIQ).

Attachment pattern	Curiosity *r* (*P* value)	Avoidance *r* (*P* value)
Secure-autonomous	.537 (.017)	−.017 (NS)
Insecure-dismissing	−.405 (.085)	−.325 (NS)
Insecure-preoccupied	.181 (NS)	.372 (NS)
Insecure-disorganized	−.231 (NS)	.642 (.003)

*Note:* NS = not statistically significant.
